# The presence and prognostic impact of apoptotic and nonapoptotic disseminated tumor cells in the bone marrow of primary breast cancer patients after neoadjuvant chemotherapy

**DOI:** 10.1186/bcr3496

**Published:** 2013-10-08

**Authors:** Andreas Daniel Hartkopf, Florin-Andrei Taran, Markus Wallwiener, Carsten Hagenbeck, Carola Melcher, Natalia Krawczyk, Markus Hahn, Diethelm Wallwiener, Tanja Fehm

**Affiliations:** 1 Department of Obstetrics and Gynecology, University of Tuebingen, Calwer Strasse 7, 72076 Tuebingen, Germany; 2 Department of Obstetrics and Gynecology, University of Heidelberg, Im Neuenheimer Feld 110, Heidelberg, Germany; 3 Department of Obstetrics and Gynecology, University of Duesseldorf, Moorenstrasse 5, 40225 Duesseldorf, Germany

## Abstract

**Introduction:**

Neoadjuvant systemic therapy of primary breast cancer (PBC) patients offers the possibility to monitor treatment response. However, patients might have metastatic relapse despite achieving a pathologic complete response (pCR). This indicates that local response to therapy must not be representative for systemic treatment efficacy. Therefore, the aim of this study was to compare local response with systemic tumor cell dissemination by determining the presence of disseminated tumor cells (DTCs), including apoptotic tumor cells, in the bone marrow (BM) of PBC patients after neoadjuvant chemotherapy (NACT).

**Methods:**

DTCs were detected by immunocytochemistry (pancytokeratin antibody A45-B/B3) and cytomorphology (DTC status). The presence of apoptotic tumor cells was determined by using the M30 antibody (M30 status). This antibody detects a neo-epitope that is expressed only during early apoptosis.

**Results:**

BM aspirates from 400 PBC patients that had completed NACT were eligible for this study. Of these, 167 (42%) patients were DTC positive (DTC status). The M30 status was investigated in 308 patients. Apoptotic (M30-positive) tumor cells were detected in 89 (29%) of these. Whereas the DTC status was not correlated (*P* = 0.557) to local treatment response (that is, pCR or a clinical complete/partial response), the presence of M30-positive tumor cells was significantly higher in patients that responded to therapy (*P* = 0.026). Additionally, DTC-positive patients were at an increased risk for disease relapse (hazard ratio, 1.87; 95% CI, 1.11 to 3.15; *P* = 0.019).

**Conclusion:**

The presence of DTC is independent of therapy response of the primary tumor. As patients that are DTC positive after NACT have an unfavorable outcome, they might benefit from additional systemic treatment.

## Introduction

Neoadjuvant chemotherapy (NACT) aims to reduce tumor mass and has become a standard treatment in primary breast cancer (PBC) patients. Initially, it was used to treat locally advanced and nonoperable tumors. Currently, NACT is also offered to patients with resectable tumors, as no differences between NACT and adjuvant treatment in terms of overall survival and the probability of disease relapse are evident [1,2]. In these patients, NACT aims to facilitate breast-conserving surgery. Moreover, NACT offers the possibility of monitoring the primary tumor’s response to treatment. This *in vivo* chemosensitivity testing helps clinicians to choose for appropriate therapy options in case of recurrent disease [3].

Breast cancer might relapse even years after successful treatment of the primary tumor. Accordingly, the disease must have the ability to persist in secondary sites of the body, a phenomenon that is called minimal residual disease (MRD). To prevent metastatic regrowth effectively, MRD has to be eradicated before it becomes clinically evident [4,5]. Monitoring of the treatment success against MRD would therefore improve therapy and the prognosis of PBC patients.

During NACT, the success of systemic treatment is currently determined locally (that is, by reassessing the primary tumor size). It is, however, not clear whether therapy response of the primary tumor is representative for the entire tumor burden (that is, for systemic treatment efficacy). Although pathologic complete response (pCR) of the primary tumor is associated with a favorable prognosis [6-9], subgroups of patients seem to not have a prognostic benefit from achieving a pCR. Minckwitz et al. recently evaluated the clinical value of pCR as related to intrinsic breast cancer subtypes and found that the primary tumor’s response to NACT has no impact on prognosis in luminal A as well as in luminal B/HER2-positive patients [10]. Other reports indicate that 15% to 25% of all patients receiving NACT will develop metastatic disease despite a pCR [11-15]. Therefore, the persistence of MRD after NACT may be independent of the success of primary tumor treatment.

It has been hypothesized that disseminated tumor cells (DTCs) in the bone marrow (BM) of PBC patients are a surrogate of MRD [16]. DTCs are detected in 13% to 40% of the cases and are an independent predictor of poor prognosis [17-25]. In addition, several studies have indicated that the presence of DTCs after adjuvant therapy predicts an increased risk of disease relapse [26-31]. However, only few smaller studies have investigated the fate and prognostic relevance of DTCs after NACT, with inconsistent results [32-35].

The aim of this study was to compare local with systemic response to NACT in a large cohort of PBC patients. For this purpose, we evaluated the prevalence of DTCs and apoptotic tumor cells at the time of surgery and compared our findings with response of the primary tumor. Moreover, we determined the prognostic relevance of DTC persistence after NACT.

## Methods

### Study population

Patients with PBC (cT1-cT4, cN0-cN2) who were treated with NACT and underwent surgery at the Department of Gynecology and Obstetrics at Tuebingen University Hospital, Germany, between January 2001 and January 2012 were eligible for this retrospective analysis. Exclusion criteria were metastatic or recurrent disease, bilateral breast cancer, and a previous history of secondary malignancy. All patients provided written informed consent for BM aspiration, and the analysis was approved by the ethics committee of the University of Tuebingen (560/2012R). Treatment regimens are shown in detail in Table 1.

**Table 1:** Neoadjuvant systemic treatment regimens

**Treatment regimen**	***n***
4 × epirubicin/cyclophosphamide (90/600 mg/m^2^ q21d)	37
4 × epirubicin/cyclophosphamide (90/600 mg/m^2^ q21d) followed by 4 × docetaxel (175 mg/m^2^ q21d)	256
4 × epirubicin/cyclophosphamide (90/600 mg/m^2^ q21d) followed by 4 × docetaxel (175 mg/m^2^ q21d) + 4 × Herceptin (6 mg/kg q21d)^a^	26
4 × epirubicin/cyclophosphamide (90/600 mg/m^2^ q21d) followed by 12 × paclitaxel (80 mg/m^2^ weekly)	7
4 × epirubicin/docetaxel (60/75 mg/m^2^ q21d)	25
6 × doxorubicin/cyclophosphamide/docetaxel (50/500/75 mg/m^2^ q21d)	29
6 × gemcitabine d1 + 8/epirubicin d1/docetaxel d1 (800/90/75 mg/m^2^ q21d)	20

^a^Loading dose: 8 mg/kg.

### Determination of response to treatment

Pathologic complete response (pCR) was defined as the absence of residual invasive cancer on pathologic evaluation of resected breast specimens and lymph nodes [36]. For patients that did not achieve a pCR, response to treatment was determined by physical examination and imaging tests. The preferred imaging modality was ultrasound. However, if ultrasound appeared not to provide valid results, other imaging tests were used, with the following priority: MRI, mammography. The effect of NACT was graded according to the World Health Organization criteria [37]. Partial remission was defined as a reduction of the primary tumor area by 50% or more (multiplication of longest diameter by the greatest perpendicular diameter; in patients with multifocal or multicentric disease, the lesion with the largest diameters was used), and also includes patients with clinical complete remission (disappearance of all known disease) that did not achieve a pCR. Progressive disease was defined as the development of new, previously undetected lesions or an increase in the diameter product of a preexisting lesion by more than 25% after at least two treatment cycles. Stable disease was defined as anything between partial remission and progressive disease. To determine response to NACT, we defined patients with pCR or partial remission as responders, whereas those with stable or progressive disease were defined as nonresponders.

### Bone marrow status and immunohistochemistry

Approximately 3 to 4 weeks after NACT, 10 to 20 ml of BM aspirates was collected during surgery. All samples were processed within 24 hours, as described elsewhere [38]. In brief, mononuclear cells were separated with density centrifugation (Ficoll, 1.077 g/ml; Biochrom, Germany), spun down onto a glass slide (Hettich cytocentrifuge; Germany) and fixed in 4% formalin. The presence of DTC (DTC status) was evaluated by immunostaining with the DAKO Autostainer (Dako, Denmark), the monoclonal mouse A45-B/B3 antibody directed against pancytokeratin (Micromet, Germany), and the DAKO-APAA detection kit (Dako). For each patient, two slides (2 × 10^6^ cells) were automatically scanned by using the ACIS imaging system (ChromaVision; Medical Systems Inc., San Juan Capistrano, CA, USA) and evaluated based on consensus recommendations for standardized tumor-cell detection and the criteria of the European ISHAGE Working group [39,40]. An additional slide was stained by using an unspecific isotype-matched antibody. Moreover, with each batch of samples, leukocytes from healthy volunteers served as negative control, and the cell lines MCF-7 and SKBR-3 were used as positive control.

To evaluate the specificity of our method for DTC-detection, we analyzed BM samples of 100 individuals without evidence of malignant disease for the presence DTCs, earlier [28]. Of these patients, one harbored DTCs.

To detect apoptotic tumor cells (M30 status), additional slides were stained with the M30-antibody (Roche Applied Science, Mannheim, Germany) and analyzed by using the previously described detection method. The M30-antibody is directed against an epitope expressed only after caspase cleavage of CK18 during early apoptosis [41,42]. Identification of apoptotic tumor cells was based on positive M30-staining and cytomorphologic criteria, as described elsewhere [34,43,44].

### Statistical analysis

Associations between categoric variables (DTC status/M30 status and patient characteristics) were analyzed by using the χ^2^ test. To determine survival, times from BM aspiration to any recurrence of disease (disease-free survival, DFS) and to death of any cause (overall survival, OS), respectively, were investigated separately. If no event occurred, data were censored at last follow-up. The influence of the DTC status/M30 status on survival was determined in a univariate analysis and described by hazard ratio (HR) and the corresponding 95% confidence interval (CI). Kaplan-Meier curves were plotted and compared with the log-rank test. For multivariate analysis, a Cox proportional regression model was used. Variables were entered stepwise backward, and a significance level of 0.1 was used to exclude a variable from the model. The initial model included menopausal status, histology, grading, nodal status before/after NACT, tumor size before/after NACT, ER/PR/HER2 status, and the DTC status. The effect of each variable was evaluated by using the Wald test and described by HR and the corresponding 95% CI. All statistical tests were carried out with PASW Statistics 20 (SPSS Inc., Chicago, IL, USA) and reported two-sided with significance levels set to *P* < 0.05.

## Results

### Patient characteristics

In total, 400 patients were included in the analysis. Details of patient characteristics are presented in Table 2. The median age was 49 (range, 21 to 87) years, and most women were premenopausal (229, 57%). The predominant histology was invasive ductal carcinoma (324, 81%). Most patients had a tumor grade II (260, 65%). Clinical tumor size before NACT was available in 385 patients, cT1-2 in 208 patients (53%) and cT3-4 in 177 patients (46%). The clinical nodal status before NACT was available in 376 patients, negative in 106 patients (28%) and positive in 270 patients (72%). After NACT, 90 patients (23%) had ypT0 tumors, 169 patients (42%) had ypT1 tumors, and 141 patients (35%) had ypT2 to 4 tumors. The nodal status after NACT was ypN0 in 197 (49%) and ypN1-3 in 203 patients (51%); 253 patients (63%) were ER-positive, 263 were PR-positive (66%), and 306 were HER2-negative (77%). 373 patients (93%) responded to NACT (75 patients had a pCR and 298 patients a partial remission), and 27 patients (7%) were nonresponders (stable and progressive disease was observed in 19 and eight patients, respectively).

**Table 2:** Patient characteristics and the prevalence of DTC and apoptotic (M30-positive) tumor cells

	**DTC**		**Apoptotic tumor cells**	
	***n*/total (%)**	***P *** **value**	***n*/total (%)**	***P *** **value**
**Total**	167/400 (42)		89/308 (29)	
**Menopausal status**				
Premenopausal	100/229 (44)		54/176 (31)	
Postmenopausal	67/171 (39)	0.368	35/132 (27)	0.425
**Histology**				
Invasive ductal	130/324 (40)		81/258 (31)	
Invasive lobular	32/66 (49)		7/44(16)	
Other	2/6 (33)		0/3 (0)	
Missing	3/4 (75)	0.418^d^	1/3 (33)	0.060^d^
**Tumor grade**				
Grade 2	105/260 (40)		51/197 (26)	
Grade 3	56/120 (47)		32/94 (34)	
Missing	6/20 (30)	0.249^d^	6/17 (35)	0.150^ d^
**Tumor size before NACT**				
cT1-2	88/208 (42)		46/167 (28)	
cT3-4	78/177 (41)		40/128 (31)	
Missing	6/15 (40)	0.833^ d^	3/13 (23)	0.488^ d^
**Nodal status before NACT**				
cN0	41/106 (39)		14/75 (19)	
cN1	115/270 (43)		70/213 (33)	
Missing	11/24 (26)	0.488 ^d^	5/20 (25)	0.020 ^d^
**Tumor size after NACT**				
ypT0	32/90 (36)		25/74 (34)	
ypT1	67/169 (40)		40/133 (30)	
ypT2-4	68/141 (48)	0.125	24/101 (24)	0.325
**Nodal status after NACT**				
ypN0	76/197 (39)		43/149 (29)	
ypN1-3	91/203 (45)	0.205	46/159 (29)	0.989
**ER status**				
Negative	67/147 (46)		42/118 (36)	
Positive	100/253 (40)	0.237	47/190 (25)	0.041
**PR status**				
Negative	67/137 (49)		43/105 (41)	
Positive	100/263 (38)	0.036	46/203 (23)	0.001
**HER2 status**				
Negative	133/306 (43)		69/237 (29)	
Positive	34/94 (36)	0.210	20/71 (28)	0.878
**Response to NACT**				
Responder^b^	156/373 (42)		87/285 (31)	
Nonresponder^c^	11/27 (41)	0.912	2/23 (9)	0.026
**Pathologic remission**				
pCR	26/75 (35)		18/60 (30)	
No pCR	141/325 (43)	0.168	71/248 (29)	0.833
**HER2-directed NACT**^**a**^				
No	24/68 (35)		8/48 (17)	
Yes	10/26 (39)	0.775	12/23 (52)	0.002

NACT, neoadjuvant systemic therapy. ^a^Only HER2-positive patients were taken into account. ^b^pCR or partial response. ^c^Stable or progressive disease.

^d^*P* value (χ^2^ test) not including missing data.

### Prevalence of DTCs and apoptotic tumor cells

After NACT, DTCs were detected in the BM of 167 (42%) patients (Table 2). A positive DTC status was observed more frequently in PR-negative patients (49%) than in PR-positive patients (38%) (*P* = 0.036). Except for the PR status, no significant association was noted between the DTC status and any established prognostic factor. Importantly, the presence of DTC was not reflected by the primary tumor response to NACT (*P* = 0.912).

BM samples of 308 patients were additionally screened for M30-positive apoptotic tumor cells. M30-positive cells were detected in 89 (29%) of these patients (Table 2). A significant association was found between the M30 status and the ER (*P* = 0.041) and PR status (*P* = 0.001), respectively. Also, patients that were nodal positive before NACT were more likely to be M30 positive (*P* = 0.020). Moreover, the prevalence of apoptotic tumor cells was significantly higher in patients that had received NACT including trastuzumab (*P* = 0.002). Importantly, there was a significant association between the presence of apoptotic tumor cells and response to NACT (*P* = 0.026).

As demonstrated in Table 3, 12 of 308 patients (4%) harbored apoptotic tumor cells only (M30-positive/DTC-negative), whereas in 77 of 308 patients (25%) both, apoptotic tumor cells and DTC, were detected (M30 positive/DTC positive). All 12 M30-positive/DTC-negative patients responded to NACT. Of the 77 M30-positive/DTC-positive patients, 75 (97%) responded to NACT. No tumor cells (M30-negative/DTC-negative) were detected in 179 of 308 patients (58%), and only DTC (M30-negative/DTC-positive) in 40 of 308 patients (13%). Of these patients, 164 (92%) and 34 (85%), respectively, responded to NACT. A subgroup analysis revealed that the presence of apoptotic tumor cells was significantly associated with response to NACT, not only in the whole collection, but also in the subgroup of DTC-positive patients (75 of 109 (69%) DTC-positive responders were M30-positive, whereas only two of eight (25%) DTC-positive nonresponders were M30-positive; *P* = 0.012).

**Table 3:** Response to NACT as related to the detection of DTCs and apoptotic (M30-positive) tumor cells

**Apoptotic tumor cells**	**DTC**	**Total**	**Responder**^**a**^	**Nonresponder**^**b**^
		***n*** **= 308**	***n *** **(%) = 285 (92)**	***n *** **(%) = 23 (8)**
+	-	12	12 (100)	0 (0)
+	+	77	75 (97)	2 (3)
-	-	179	164 (92)	15 (8)
-	+	40	34 (85)	6 (15)

(*P* = 0.068, χ^2^ test). ^a^pCR or partial response; ^b^stable or progressive disease.

### Survival analysis

Follow-up data for the calculation of DFS and OS were available in 330 and 376 patients, respectively. The median follow-up was 45.34 months for DFS and 54.46 months for OS. Univariate analysis revealed no impact of the M30 status on DFS (*P* = 0.162) and OS (*P* = 0.097). Also, in a subgroup analysis regarding only DTC-positive patients, the M30status was associated neither with DFS (*P* = 0.402) nor with OS (*P* = 0.152).

**Figure 1:**
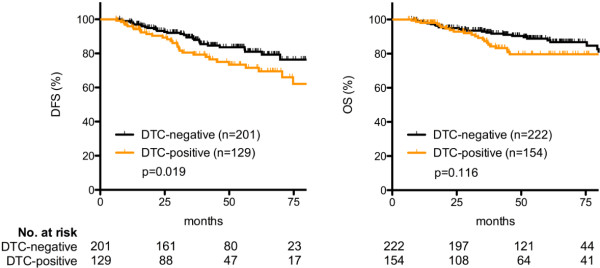
Disease-free (DFS) and overall (OS) survival of DTC-negative versus DTC-positive patients.

There was a significant difference between DFS of DTC-negative versus DTC-positive patients (Figure 1): 30 events occurred in the group of 201 DTC-negative patients and 31 events in the group of 129 DTC-positive patients (HR, 1.87; 95% CI, 1.11 to 3.15; *P* = 0.019). However, the DTC status had no influence on OS (27 events occurred in the group of 222 DTC-negative patients, and 25 events, in the group of 154 DTC-positive patients, *P* = 0.116).

In multivariate analysis (Table 4), independent factors of DFS were DTC status, nodal status after NACT, and response to NACT. Independent factors of OS were tumor grade and response to NACT.

**Table 4:** Multivariate Cox regression analysis of survival

	**DFS**		**OS**	
	**HR**	***P *** **value**	**HR**	***P *** **value**
	**(95% CI)**		**(95% CI)**	
**DTC status**				
Positive versus negative	1.87 (1.09 to 3.20)	0.027	NS	
**Tumor grade**				
Grade III versus Grade II	NS		3.04 (1.66 to 5.55)	<0.001
**Nodal status after NACT**				
ypN1-3 versus ypN0	2.22 (1.18 to 4.19)	0.014	NS	
**Response to NACT**				
Nonresponder^a^ versus				
responder^b^	2.88 (1.32 to 6.31)	0.008	2.42 (1.04 to 5.64)	0.041

PFS, *progression-free survival;* OS *overall survival;* CI, *confidence interval;* NS, *not significant;* HR, hazard ratio. ^a^Stable or progressive disease; ^b^pCR or partial response.

## Discussion

In the past decade, NACT has become an increasingly important strategy to treat PBC patients. Many clinical trials on systemic breast cancer treatment are currently conducted in the neoadjuvant situation, by using response to therapy as an early and easy-to-perform study end point and a surrogate for prognosis. Although response to NACT is associated with disease-free and overall survival some subgroups of patients do not benefit from achieving pCR and approximately 15% to 25% of the patients may develop metastatic disease despite pCR [10-15]. This indicates that the disease must have the ability to persist in secondary sites of the body and that local response to therapy is not necessarily representative of systemic response. Because micrometastatic spread of DTCs into the BM is a promising marker of disease persistence, this large retrospective analysis aimed to compare local response to NACT, as reflected by the primary tumor, with systemic response, as reflected by the presence of DTC and apoptotic tumor cells. We moreover determined the impact of the DTC status after NACT on prognosis.

DTCs were detected in 42% of the patients. An explanation for this comparatively high prevalence is that most women had initially advanced stages with nodal involvement. In general, these patients tend to have higher positivity rates in BM aspirates [17,18,20-22,34,45]. Importantly, the presence of DTCs after NACT was not reflected by response of the primary tumor, indicating that local response to treatment is independent of systemic treatment efficacy. This observation is in line with previous reports and most likely due to altered genomic characteristics between CTC/DTC and primary tumor tissue of the same patient [32,33,35,46]. For example, phenotypic changes like HER2-amplification or epithelial-to-mesenchymal transition (EMT) can occur during tumor cell dissemination [47,48]. Moreover, a large proportion of DTCs in breast cancer patients display stem cell-like features, such as ALDH1 positivity or presence of CD44 and absence of CD24 [49]. The cancer stem-cell hypothesis postulates that these cells not only might initiate tumorigenesis and metastatic growth, but also contribute importantly to therapy resistance [50].

Apoptosis is the principal mechanism of chemotherapy-induced tumor regression, and several studies have reported on the detection of single apoptotic cells in either peripheral blood or BM of breast cancer patients [34,51-53]. To detect apoptotic cells, we and others used immunocytochemical staining with the M30-antibody [34,51]. The neo-epitope M30 is expressed by caspase cleavage of CK18 only during early apoptosis. Interestingly, serum levels of the CK fragment were shown to correlate with tumor load and prognosis during breast cancer chemotherapy [54]. In line with our previously presented results, the presence of apoptotic tumor cells was associated with local therapy response [34].

To differentiate between DTCs and false-positive cells, morphologic evaluations were performed as recommended by the ISHAGE Working Group. Therefore, cells with typical morphologic signs of apoptosis (cellular shrinkage, membrane blebbing, nuclear condensation, and fragmentation) were not considered as DTCs. As the majority of DTC-positive patients also harbored M30-positive cells, we believe that tumor cells in the BM represent a heterogeneous population of apoptotic and nonapoptotic cells. However, we cannot exclude that some DTCs might be M30 positive (that is, apoptotic), as the M30 status was evaluated on additional slides, and a double-staining procedure was not performed for technical reasons. Moreover, A45-B/B3 detects CK8, CK18, and CK19, whereas M30 detects a neoepitope of CK18, only. Thus, differences in the results between these two antibodies might be biased by different CK-expression patterns.

Principally, two different mechanisms would explain the source of apoptotic tumor cells in BM aspirates. These could be the result of a passive cell shedding from the primary tumor during NACT [35] and therefore reflect local response to NACT. The association between local treatment response and the M30 status we observed supports this hypothesis. Also, advanced tumor stage and proliferation are associated with an increased tumor-cell turnover, and a higher breast cancer grade was recently shown to result in increased shedding of apoptotic tumor cells into the circulation [51,55]. In accordance, we found that patients at advanced stages (nodal positive) before the beginning of NACT as well as hormone-receptor-negative patients were more likely to present with apoptotic tumor cells in BM.

Conversely, DTCs that are the result of an active premetastatic process might undergo apoptosis, caused by systemic efficacy of NACT. However, chemotherapy is often unable to eradicate nonproliferating DTCs [27]. Indeed, we could not find an association between the presence of apoptotic cells and survival. A subgroup analysis, regarding the impact of the M30 status on survival in DTC-positive patients, confirmed this observation. Some authors therefore suggested the use of bisphosphonates as an alternative for cell-cycle-independent treatment of DTC-positive patients [56-58]. Others indicate that targeted therapy might be more appropriate than conventional chemotherapeutic regimens to eradicate MRD successfully [27,59,60]. Interestingly, we found that the prevalence of apoptotic tumor cells in patients that received trastuzumab was increased. This indicates a specific action of HER2-directed therapy against a significant subpopulation of DTCs. We recently found, in a large cohort of PBC patients, that about half of the DTC-positive patients harbor HER2-positive DTCs, independent of the HER2 status of the primary tumor [48]. The clinical value of HER2-directed therapy to treat MRD will be evaluated within the forthcoming “TREAT CTC” study (http://NCT01548677). In this trial, HER2-negative PBC patients with one or more CTCs after (neo)adjuvant chemotherapy will be randomized to receive trastuzumab treatment or not.

In PBC patients that have not been treated systemically before BM aspiration, the detection of DTCs has clearly proven to be of prognostic relevance, whereas the role of DTC detection after NACT is less well described [17-25,32,33]. Hall *et al.*[33] recently conducted a study on 95 PBC patients that were treated with neoadjuvant chemotherapy and found that DTC persistence is associated with breast-cancer-specific survival. Mathiesen *et al*. [32] evaluated the presence of DTC in the BM before, directly after (at surgery), and 12 months after NACT [32]. Probably due to the considerably low number of patients (*n* = 69), the authors found no impact of the DTC status directly after NACT on survival. In contrast to the results presented by Mathiesen *et al*., our study demonstrates an independent prognostic value of DTC detection after NACT. Of note, it would be interesting to evaluate the prognostic relevance of DTC persistence in patients with luminal B/HER2-positive or luminal A tumors separately, as pCR is not predictive of survival in these subgroups [10]. However, the number of patients was too low to permit such an analysis.

Next to the herein investigated role of the DTC status after NACT, promising data exist on DTC persistence in PBC during or after adjuvant treatment [28,29,31,32]. As these studies could also demonstrate an independent prognostic impact in patients that have received systemic therapy, the DTC status might be a marker of treatment failure and is likely to indicate patients that are in need of additional treatment. In the Norwegian SATT study (NBCG9), 72 PBC patients that were DTC positive after anthracycline-containing adjuvant chemotherapy received secondary docetaxel treatment, and a majority (79%) experienced disappearance of DTCs [61]. However, the clinical relevance of these results is not clear, as follow-up is still ongoing.

We could not find a prognostic impact of DTC determination after NACT on OS. Interestingly, a subgroup analysis revealed that in pretherapeutic clinical tumor stage III patients, the DTC status was prognostic for DFS and also OS, whereas in stage I to II patients, no impact of the DTC status on survival was found (data not shown). This observation is in accordance with our recent analysis on 1,345 clinically nodal-negative PBC patients [62]. In that study, the DTC status had also no impact on survival.

As BM sampling is a comparatively invasive procedure, recent reports have focused on the detection of CTC in the peripheral blood of PBC patients [63-65]. Whereas CTC detection in metastatic breast cancer patients has proven to be of prognostic relevance [66-68], their role in PBC is less well described, and results on an association between CTC and response to NACT or prognosis are inconclusive [60,69-71]. This is probably owing to methodologic differences and a lower sensitivity of CTC analysis [25]. Recently, Molloy *et al.*[72] found, in 733 PBC patients, that CTC detection by use of a PCR-based assay was highly associated with the presence of DTCs, and that both DTC and CTC detection were predictive of survival. Further trials should therefore evaluate the clinical value of DTC/CTC enumeration in the neoadjuvant situation, especially as CTC detection offers the possibility of serial blood sampling during the course of therapy.

## Conclusions

The detection of DTCs in the bone marrow of PBC patients is considered as a marker of systemic disease. In line with recent smaller studies, we found that the presence of DTCs after NACT was independent of the primary tumor response to treatment. As the DTC status was moreover associated with an unfavorable outcome, even patients with pCR but DTC persistence after NACT might benefit from additional adjuvant therapy. Moreover, tumor cells in BM seem to represent a heterogeneous population of apoptotic and nonapoptotic cells. Although the prevalence of apoptotic tumor cells was increased in patients that responded to NACT, no influence of apoptotic tumor cell detection was found on prognosis. The clinical relevance of monitoring apoptosis during therapy remains unclear. Further characterization of DTCs and clinical trials are needed to understand the biologic mechanism of tumor cell persistence and to determine their impact on optimizing (neo)adjuvant breast cancer treatment.

## Abbreviations

BM: Bone marrow; CI: Confidence interval; CK: Cytokeratin; CTC: Circulating tumor cell; DFS: Disease-free survival; DTC: Disseminated tumor cell; ER: Estrogen-receptor; HER2: Human epithelial growth factor receptor 2; HR: Hazard ratio; MRD: Minimal residual disease; NACT: Neoadjuvant systemic therapy; NS: Not significant; OS: Overall survival; PBC: Primary breast cancer; pCR: Pathologic complete response; PR: Progesterone receptor.

## Competing interests

All authors declare that they have no conflict of interest.

## Authors’ contributions

AH designed the study, made substantial contributions to interpretation of data, and drafted the manuscript. FT made substantial contributions to acquisition of data and critically revised the manuscript for important intellectual content. MW and CH made substantial contributions to conception and design of the study and helped to draft the manuscript. CM made substantial contributions to interpretation of data and helped to draft the manuscript. NK and MH made substantial contributions to acquisition of data. DW made substantial contributions to conception and design of the study and critically revised the manuscript for important intellectual content. TF designed the study, performed the statistical analysis, and helped to draft the manuscript. All authors read and approved the final manuscript.
